# Integrative taxonomy of a new *Redudasys* species (Gastrotricha: Macrodasyida) sheds light on the invasion of fresh water habitats by macrodasyids

**DOI:** 10.1038/s41598-018-38033-0

**Published:** 2019-02-14

**Authors:** André R. S. Garraffoni, Thiago Q. Araújo, Anete P. Lourenço, Loretta Guidi, Maria Balsamo

**Affiliations:** 1 Department of Animal Biology, Institute of Biology, State University of Campinas, R. Monteiro Lobato, 255, 13083-970 Campinas, SP Brazil; 2 Departament of Zoology, Institute of Biological Science, Federal University of Minas Gerais, Av. Antonio Carlos, 6627, 31270-901 Belo Horizonte, MG Brazil; 3 Departament of Biological Science, Federal University of Jequitinhonha and Mucuri Valleys, Rod. BR-367, 39100-000 Diamantina, MG Brazil; 4 Department of Biomolecular Sciences, University of Urbino, Campus Scientifico, Via Ca’ le Suore, 2, 61049 Urbino, PU Italy

## Abstract

The order Macrodasyida (Gastrotricha) includes over 350 marine species, and only 3 freshwater species (*Marinellina flagellata*, *Redudasys fornerise, R*. *neotemperatus*). Herein we describe a new freshwater species of Macrodasyida, *Redudasys brasiliensis* sp. nov., from Brazil through an integrative taxonomic approach. The external morphology and internal anatomy were investigated using differential interference contrast microscopy, confocal microscopy, scanning and transmission electron microscopy. The systematization of the new taxon was inferred by nuclear (18S and 28S) and mitochondrial (COI) genes, and its intra-order relationships were assessed using data from most of available macrodasyids. Phylogenetic analyses yielded congruent trees, in which the new taxon is nested within the family Redudasyidae, but it was genetically distinct from the other species of the genus *Redudasys*. The new species shares the gross morphology and reproductive traits with other Redudasyidae and the presence of only 1 anterior adhesive tube per side with *Redudasys neotemperatus*, but it has a specific pattern of ventral ciliation and muscle organization. Results support the hypothesis that dispersion into fresh water habitats by Macrodasyida and Chaetonotida taxa occurred independently and that within Macrodasyida a single lineage invaded the freshwater environment only once. Furthermore, the Neotropical region seems to be peculiar for the evolution of the freshwater macrodasyid clade.

## Introduction

Gastrotricha is a group of aquatic, free-living microinvertebrates (80 µm to 3,000 µm in body length) divided into two orders: Macrodasyida and Chaetonotida^1–4^. The taxon Macrodasyida comprises over 350 worm-like species^5^, all interstitial in marine and estuarine habitats, except *Marinellina flagellata* Ruttner-Kolisko, 1955 collected from Austria, *Redudasys fornerise* Kisielewski, 1987 from Brazil and *R. neotemperatus* Kånneby & Kirk, 2017 from the U.S.A.^6–9^. However, at least two other unidentified freshwater macrodasyid species were collected from Brazil (*Marinellina* sp.^10^ and *Redudasys* sp.^11^).

Ruttner-Kolisko^6^ described for the first time a new freshwater species belonging to the order Macrodasyida, *Marinellina flagellata*, from the hyporheic region of an Austrian river, based on two likely immature specimens (the author reported that she could not see the reproductive organs). More than thirty years later Kisielewski^7^ described *Redudasys fornerise*, the first undoubted taxon of the order Macrodasyida recorded from a freshwater reservoir in Brazil. Todaro *et al*.^8^ redescribed the latter species few years ago. Recently, Garraffoni *et al*.^11^ found many specimens belonging to the genus *Redudasys* showing some minor differences from *Redudasys fornerise*, and Araújo *et al*.^10^ reported the occurrence of an undescribed species provisionally assigned to the genus *Marinellina* in South America for the first time. Furthermore, Kånneby & Wicksten^12^ collected one specimen of a putative new species of *Redudasys* from the Edwards Aquifer, Texas (U.S.A.): that was the first record of the genus from the Nearctic biogeographic region. Three years later, Kånneby & Kirk^9^ formally described the unnamed species reported by Kånneby & Wicksten^12^ as *Redudasys neotemperatus* based on specimens collected in Oregon (U.S.A.). An additional finding of a macrodasyid gastrotrich specimen much likely belonging to *Marinellina flagellata* was made by J.M. Schmidt-Araya in a different Austrian stream (Todaro *et al*.^8^).

Integrative taxonomy is a new approach for taxonomic and systematic studies that has spread in recent years^13–15^. The basic idea is to integrate information from different sources: ecological data, molecular data from nuclear and mitochondrial DNA, and morphological characters. In order to better understand the evolutionary history of the studied taxa, the data integration can be done by cumulation or congruence frameworks^13^. In the former case, the species identification is performed by a cumulative set of characters, without necessarily requiring concordance among all of them. Thus, a single set of characters considered good can be used to support the hypothesis about the new species. On the other hand, the framework of integration by congruence is based on a lineage divergence and the hypothesis under the assumption is based on the integration or coherent pattern of two or more independent sources of characters^13^. Considering that the advent of digital techniques in optical and electron microscopy have speed up the finding of new morphological characters and that new cybernetic infrastructures have been developed for analyzing and integrating data, the documentation and dissemination of data have greatly been improved^16^.

Herein, we formally describe a new species of freshwater Macrodasyida from Brazil through the approach of integrative taxonomy with the aim to investigate the relationships of the new taxon within the order. Morphological techniques (DIC, CLSM, SEM, TEM) and multigene molecular analyses (18S rRNA, 28S rRNA and COI mtDNA) were applied. We chose to integrate the data examined in this study by congruence: this framework has high confidence to promote taxonomic stability because the hypothesis regarding the description of the new species is supported by several character sets^13^. A study of additional specimens of *Redudasys fornerise* allowed a comparison within the genus.

## Results

**Taxonomic account**

Phylum Gastrotricha Metschnikoff, 1865

Order Macrodasyida Remane, 1925 [Rao & Clausen, 1970]

Family Redudasyidae Todaro, Dal Zotto, Jondelius, Hochberg, Hummon, Kånneby & Rocha, 2012

Genus *Redudasys* Kisielewski, 1987

### Emended diagnosis of the family Redudasyidae

Macrodasyids from 302 to 400 µm in total length, with rounded head bearing several sensory cilia but without tentacles or ocelli. Lateral trunk margins even, without indentations or protrusions. Posterior end two-lobed, without a peduncle. Cuticular covering smooth, without scales or spines. Adhesive apparatus consisting of anterior (TbA) and posterior tubes (TbP); ventrolateral tubes (TbVL) may also be present (*Anandrodasys*). TbA, 1–3 per side: 1 tube per side arising from a lateral pouch, or 2–3 tubes of unequal length per side, borne from a common base and emerging from a ventrolateral furrow (*Redudasys*) or inserted in parallel (*Anandrodasys*), protruding obliquely to the rear. TbP, 4–12 in total, distributed symmetrically at the end of the two caudal lobes. TbVL if present at all, 5–6 per side, along the anterior intestinal region. Dorsal tubes (TbD) and lateral tubes (TbL) absent. Longitudinal muscles visibly cross-striated. Ventral ciliation of different patterns in the 2 genera: *Redudasys*: paired fields posterior to the mouth and along the pharyngeal region (U04–U36), and unpaired patches along the median line of the trunk region (U39–U50) or arranged in two large longitudinal bands close to each other, extending from the mouth ring to the anterior trunk (U48) and then merging into a single unpaired band to the caudal end; *Anandrodasys*: an unified field posterior to the mouth splits into 2 paired longitudinal bands, with the medial along the pharyngeal region, and the lateral extending up to the anus posterior to which is an isolated unpaired patch. Mouth, terminal or slightly subterminal; buccal cavity inconspicuous. Pharynx bearing pores at base, opening ventrolaterally. Intestine straight; anus ventral. Protonephridia present, 3 per side. Parthenogenetic; ovaries paired in hindgut region, with oocytes maturing anteriorly; male apparatus unknown; frontal and caudal organs unknown. Interstitial, marine or freshwater.

Type genus: *Redudasys* Kisielewski, 1987; other genus: *Anandrodasys* Todaro, Dal Zotto, Jondelius, Hochberg, Hummon, Kånneby & Rocha, 2012.

**Figure 1:**
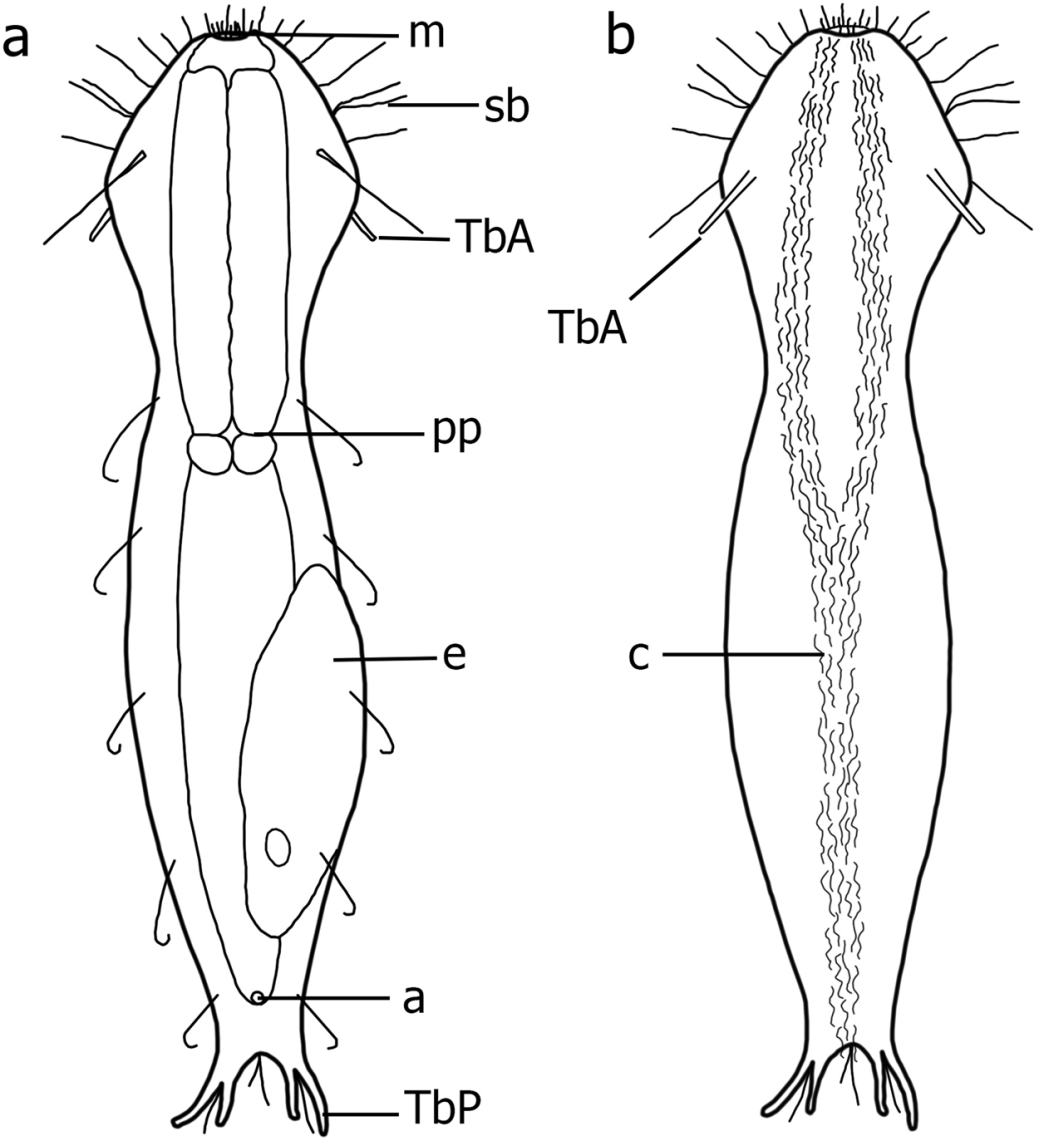
*Redudasys brasiliensis* sp. nov. Schematic drawing. (**a**) Dorsal view. (**b**) Ventral view. a = anus, c = locomotory ventral ciliation. e = egg, m = mouth, pp = pharyngeal pore, sb = sensory bristles, TbA = anterior adhesive tube, TbP = posterior adhesive tube.

**Figure 2:**
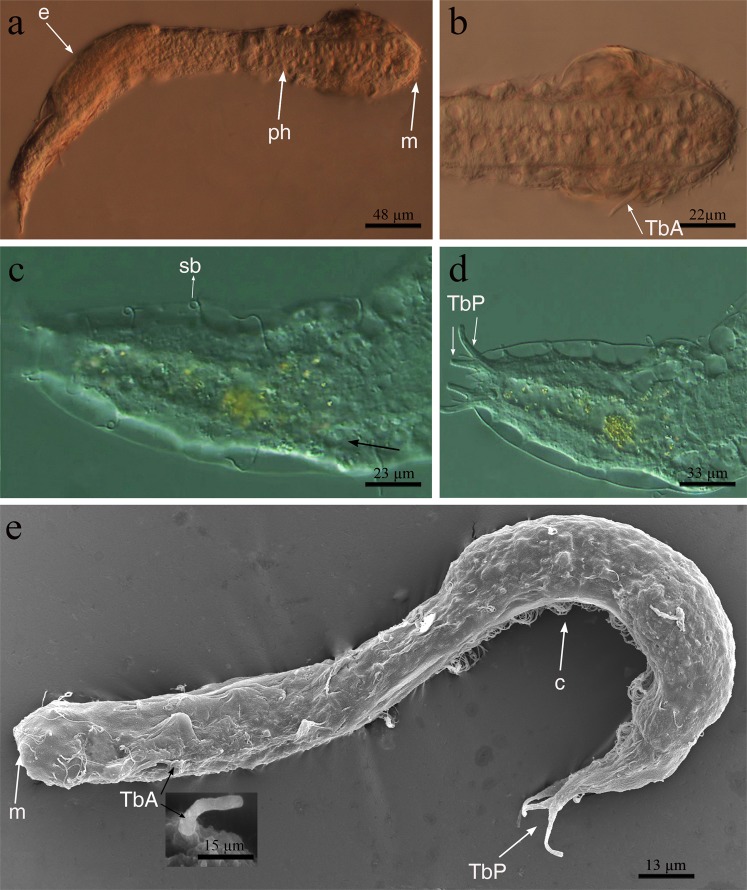
*Redudasys brasiliensis* sp. nov. Optical DIC micrographs. (**a**) Habitus. (**b**) Anterior body end. (**c**) Dorsal posterior region. (**d**) Posterior and caudal regions. Scanning electron micrographs. (**e**) Habitus in dorsolateral view and close-up of an anterior adhesive tube (inset). c = locomotory ventral ciliation, e = egg m = mouth, ph = pharynx, sb = sensory bristles, TbA = anterior adhesive tubes, TbP = posterior adhesive tubes.

**Figure 3:**
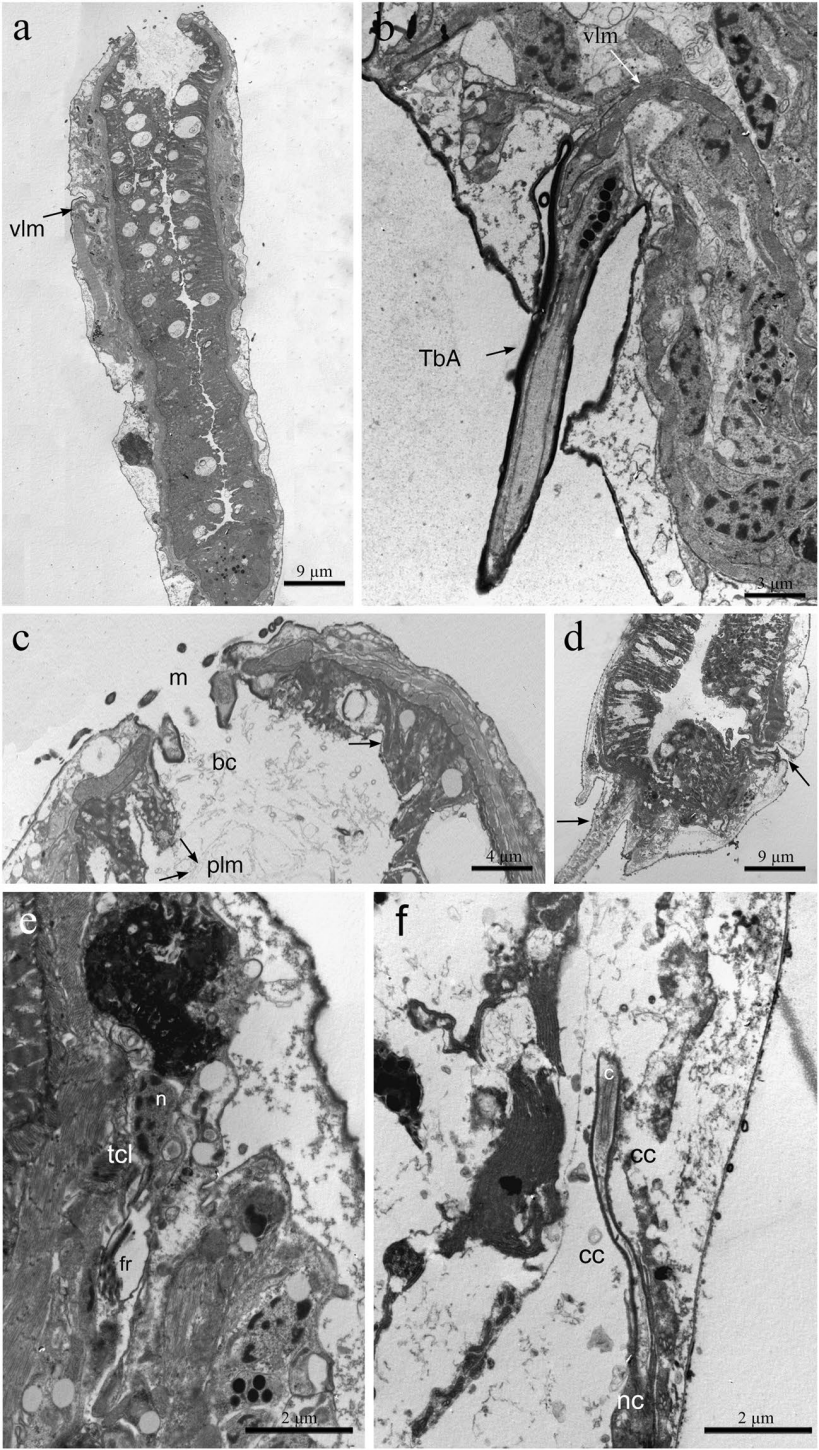
*Redudasys brasiliensis* sp. nov. Transmission electron micrographs. (**a**) Longitudinal section of the anterior body region showing the mouth and the whole pharynx. Note the ventrolateral muscles running from the lateral pouch. (**b**) Longitudinal section of an anterior adhesive tube arising from a lateral pouch; the insertion of ventrolateral muscle is visible. (**c**) Longitudinal section of anterior body end, showing the terminal mouth, the buccal cavity lined with a thin cuticle (arrow) and the pharyngeal longitudinal muscles inserted at mouth ring. (**d**) Cross-section of the pharynx, showing the Y-inverted lumen and the insertion points of the TbA (arrows). (**e**) Longitudinal sections of a protonephridium, showing the nuclear and filter region of the terminal cell. (**f**) Longitudinal sections of a protonephridium, showing, anal cell with a single luminal cilium and a nephridiopore cell. bc = buccal cavity, c = cilium, cc = canal cell, fr = filter region, m = mouth, n = nucleus, nc = nephridiopore cell, plm = pharyngeal longitudinal muscles, TbA = anterior adhesive tubes, tcl = terminal cell, vlm = ventrolateral muscles.

**Figure 4:**
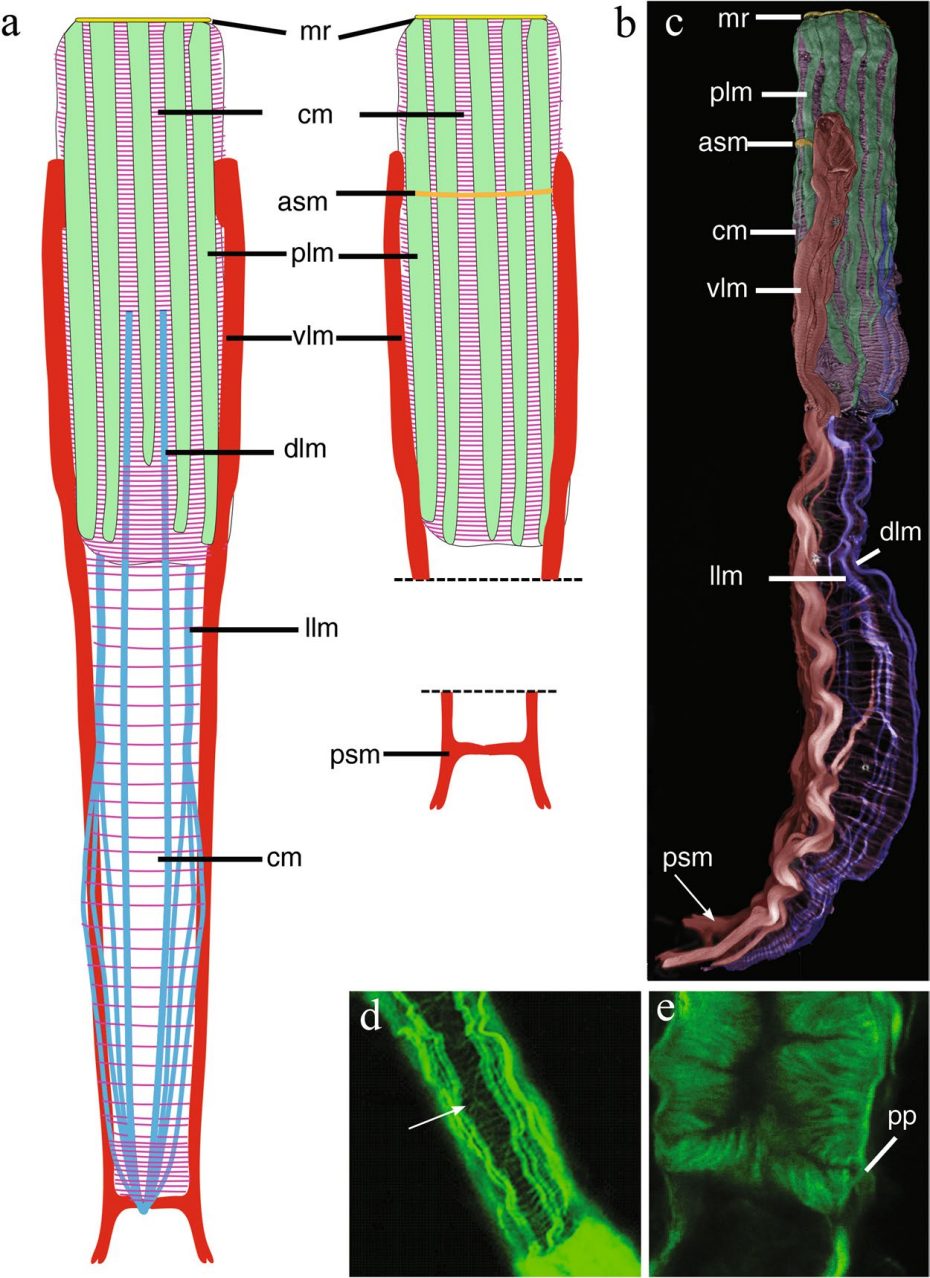
*Redudasys brasiliensis* sp. nov. Reconstruction of the musculature from confocal laser scanning microscopy images. (**a**) Schematic drawing of musculature in dorsal view. (**b**) Schematic detail of ventral musculature in the pharyngeal and the caudal regions. (**c**) Volocity-rendered 3D view of muscles in lateral view. Confocal micrographs of phalloidin-stained specimens. (**d**) Helicoidal muscles in the pharynx (arrow). (**e**) Detail of the pharynx posterior end with pharyngeal pores. asm = anterior semicircular muscle, cm = circular muscles, dlm = dorsal longitudinal muscles, llm = dorsal longitudinal muscles, mr = mouth ring, plm = pharyngeal longitudinal muscles, pp = pharyngeal pore, psm = posterior semicircular muscle, vlm = ventrolateral muscles.

*Redudasys brasiliensis* sp. nov. (Figs 1–4)

### Diagnosis

Macrodasyid 302–376 µm in total body length. Body separated into head, trunk and caudal regions. Head rounded, bearing several sensory cilia, without tentacles or ocelli. Cylindrical trunk, posterior end two-lobed, without a peduncle. Cuticular covering smooth, with no ornamentation. Paired tactile bristles of equal length along the body sides. Adhesive tubes only at the anterior and posterior body ends. TbA, only one tube on each head side, arising from a ventrolateral pouch. TbP, two symmetrical groups, each composed of two tubes of unequal length arising from a common base. TbD and TbL absent. Ventral ciliation arranged in two large longitudinal bands close to each other, extending from the mouth ring to the anterior trunk then merging into a single band up to the caudal body end. Mouth terminal; buccal cavity inconspicuous, lined with thin cuticle. Cylindrical pharynx with pharyngeal pores at base. Circular muscles surround the entire pharynx and are in turn surrounded by helicoidal muscles. Ten to twelve longitudinal muscles are external to both circular and helicoidal muscles, and extend from the mouth ring to the caudal end. Two of these muscles, the ventrolateral bands, insert anteriorly on a transverse muscle at the region of the TbA, and caudally on a second transverse muscle. Muscles of the trunk include longitudinal bands along the intestine that are surrounded by somatic circular muscles; the paired ventrolateral longitudinal muscles run along their length externally to the somatic circular muscles. Protonephridia present. Intestine straight, narrowing posteriorly; anus ventral. Parthenogenetic; paired ovaries in posterior region of intestine, with oocytes maturing anteriorly; male system absent; caudal and frontal organs absent. Freshwater, interstitial.

### Etymology

The specific name refers to the country where the species was found.

### Species-specific characters

Ventral ciliation made of 2 large longitudinal bands close each other and merging into one at the anterior trunk region. Longitudinal muscles of the trunk up to posterior end: 2 dorsal from mid-pharynx and 2 lateral branching into 3 parallel bundles; thin anterior transverse muscle connecting the two ventral longitudinal muscles.

### Material

Holotype. Adult, collected from sandy pebble-sediment on November 20, 2013, at 0.3 m depth in the Soberbo Stream, Jequitinhonha drainage basin, Diamantina, State of Minas Gerais, Brazil, mounted on glass slide, deposited at the Zoological Museum, State University of Campinas, Brazil, accession number ZUEC GCH 05.

Paratypes. Six adult specimens and one subadult specimen, collected from sandy pebble-sediment on November 19–20, 2013, at 0.3 m depth in the Soberbo Stream, Jequitinhonha drainage basin, Diamantina, State of Minas Gerais, Brazil, mounted on glass slide, deposited at the Zoological Museum, State University of Campinas, Brazil, accession numbers ZUEC GCH 06 (2 specimens), ZUEC GCH 07 (2 specimens) and ZUEC GCH 08 (2 specimens).

### Repository

urn:lsid:zoobank.org:pub:B5C8B48B-FA7E-45DE-8160-DFA80204EA06.

### Other material

Ten additional specimens collected on September 10, 2010 and on July 07, 2014 in the Água Limpa Stream, Jequitinhonha drainage basin, Diamantina, State of Minas Gerais, Brazil and two specimens collected on May 3, 2010 in an unnamed stream, São Francisco drainage basin, Sempre-Vivas Federal Park, Diamantina, State of Minas Gerais, Brazil were observed alive and are no longer available. Four were filmed and videos were deposited at the Zoological Museum, State University of Campinas, Brazil, accession numbers ZUEC GCH 51 to ZUEC GCH 54.

Three adult specimens, collected on July 07, 2014 in Água Limpa Stream, Jequitinhonha drainage basin, Diamantina, State of Minas Gerais, Brazil, were mounted for SEM, and kept at the State University of Campinas.

Four adult specimens collected on March 15, 2014 in Água Limpa stream, Jequitinhonha drainage basin, Diamantina, State of Minas Gerais, Brazil, were prepared for TEM and are no longer available.

Three adult specimens collected on August 20, 2015 in Água Limpa stream, Jequitinhonha drainage basin, Diamantina, State of Minas Gerais, Brazil, were prepared for confocal analysis and are no longer available.

Four adult specimens, collected in Veado Pool and Preto River, Jequitinhonha drainage basin, São Gonçalo do Rio Preto, State of Minas Gerais, Brazil (March 2013 and June 2015) and eight adult specimens collected in Água Limpa and Soberbo streams, Jequitinhonha drainage basin, Diamantina, State of Minas Gerais, Brazil (April 2015, February 2016 and December 2017), were used for DNA extraction and are no longer available (Supplementary Table S1).

### Description

The description is based on both the holotype and 7 paratypes (Fig. 1a,b; Table 1). Worm-like body, flattened ventrally, vaulted dorsally, 302–376 μm in total length. Body well-delimited into head/trunk/caudal regions. Head width (U17) 44–65 μm, anterior trunk width (U40) 30–64 μm, midtrunk width (U60) 33–68 μm, and caudal base width (U93) 18–30 μm. Head rounded anteriorly, without tentacles or ocelli. Caudal end bearing two symmetrical groups each of two tubes of unequal length arising from a common base.

**Table 1:** Morphometric parameters for *Redudasys brasiliensis* sp. nov. A = Average value, Holo = Holotype, Par = Paratype, SD = Standard Deviation.

Character	Holo	Par1*	Par2**	Par3	Par4	Par5	Par6	Par7	A	SD
Total body length	370	193	296	302	310	313	375	376	320	60
Pharynx length	144	65	86	125	130	147	132	145	127	26
Maximum body width at mouth	26	10	35	20	29	22	25	20	22	6
Maximum body width at TbA	62	44	86	61	58	60	65	60	59	6
Maximum body width at PIJ	46	30	64	49	41	43	46	40	42	6
Maximum body width at base of caudal end	23	18	20	21	23	26	25	23	22	3
TbA - tubes length	15	14	12	12	13	11	11	10	12	2
TbP – medial tubes length	13	9	10	10	13	15	15	13	13	2
TbP - lateral tubes length	18	14	18	18	19	19	20	21	18	2
Pharynx width	30	20	40	34	30	30	30	30	29	4

All measures in µm. * A subadult. ** Paratype 2 had the body clearly contracted due to MgCl_2_ action and was not used to calculate the A and SD.

Cuticular covering smooth, without scales, spines or epidermal glands, (Figs 2a–e and 3a,b). Protonephridia composed of 3 cells: proximal terminal cell, canal cell and distal nephridiopore cell (Fig. 3e,f).

#### Ciliation

Sensory cilia distributed irregularly, scattered around the mouth (ca.10 μm in length) and along the anterolateral head margin (ca. 30 μm in length). Six paired dorsolateral sensory bristles of equal length (ca. 20 μm in length) along the body sides and one paired bristle on each caudal appendage (Fig. 2c,d). Ventral locomotory ciliation arranged in two large longitudinal bands close to each other, extending from the mouth ring to the anterior trunk (U48) and then merging into a single band to the caudal body end (Fig. 1b).

#### Adhesive tubes

Only TbA and TbP are present (Figs 1a,b, 2b,d and 3b). TbA, 1 per side, 15 μm long in the holotype, located at U16–18, and arising from a deep ventrolateral pouch (Fig. 3b,d). Lateral and dorsal tubes absent (Figs 1a,b and 2a,e). TbP, 2 per side, of unequal length, the outer 18–21 μm long and the inner 10–15 μm (Figs 1a,b and 2d,e).

#### Digestive tract

Mouth opening terminal and rounded, 10–29 μm in diameter (26 μm in the holotype). Buccal cavity inconspicuous, 14 µm long, lined with thin cuticle and supported by a strong musculature (Fig. 3a,c). Cylindrical pharynx, 65–147 μm long and 20–34 μm wide (Figs 2a and 3a). Triradial inverted-Y lumen (Fig. 3d). Poorly developed ventrolateral pores occur at the posterior end of the pharynx (U36) (Fig. 4e). Intestine slightly narrowing posteriorly, anus at ca. U90.

#### Reproductive system

Male reproductive organs not seen, species probably parthenogenetic. Two ovaries lateral to the posterior intestine (ca. U82); usually one fully-grown egg (120 μm long) is visible dorsally (Fig. 2a).

#### Muscular system

Thin circular muscles, very numerous, surround the pharynx for its whole length (U01 to U43). Circular muscles of the intestine (U44 to U82) are less numerous and more regularly spaced; muscles noticeably closer each to other at the caudal end (U83 to U93) (Fig. 4a,c). Along the pharynx, 10 to 12 large, longitudinal muscles are inserted at the mouth ring and extend to the PhJIn (U43): they get thinner posteriorly (Figs 3c and 4a–c) and are external to circular muscles. One pair of dorsal longitudinal muscles extends from the mid pharynx to the caudal end (U23-U95) (Fig. 4a,c). They are external to circular muscles, except in the posterior pharyngeal region (U31-U37) and in the caudal body region (U83-U93), where they lie beneath the circular muscles (Fig. 4a,c). One pair of lateral longitudinal muscles extend from the PhJIn (U43) and branch into 3 parallel, thin bundles on each side from U61 to the body posterior end (U93). These longitudinal muscles are surrounded by circular muscles for their whole length (Fig. 4a,c).

The largest muscles in the body are two longitudinal muscles that are inserted laterally to the pharynx in the region of the TbA (U14) and run ventrolaterally extending up to the caudal appendages (Figs 3a,b and 4a–c). These muscles are connected anteriorly (U16) by a thin ventral transverse muscle and posteriorly (U96) by another transverse semicircular muscle (Fig. 4b,c).

Helicoidal muscles were observed along the pharyngeal region, ending at the anterior intestinal region (U48) (Fig. 4d).

#### Ecology

Species relatively common in sediment of the Jequitinhonha drainage basin sites; rare in sediment of the São Francisco drainage basin sites.

*Redudasys fornerise* Kisielewski, 1987

(Figures 5, 6)

**Figure 5:**
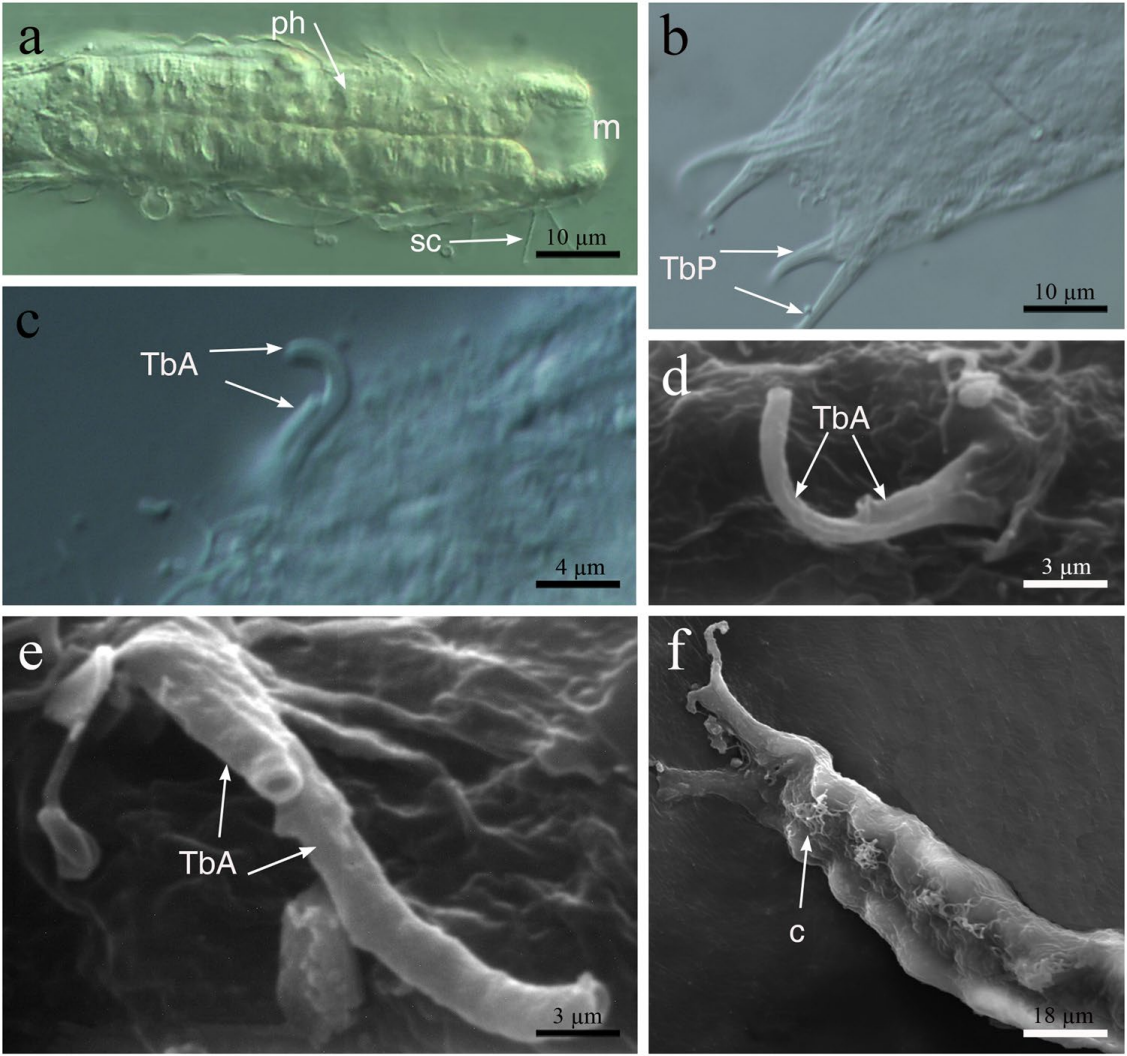
*Redudasys fornerise*. Optical DIC micrographs. (**a**) Body anterior region. (**b**) Posterior and caudal regions. (**c**) Close-up of a pair of anterior adhesive tubes. Scanning electron micrographs. (**d**,**e**) Close-up of the anterior adhesive tubes. (**f**) Ventrolateral view of the posterior region. c = locomotory ciliation, m = mouth, ph = pharynx, sc = sensory cilia, TbA = anterior adhesive tubes, TbP = posterior adhesive tubes.

**Figure 6:**
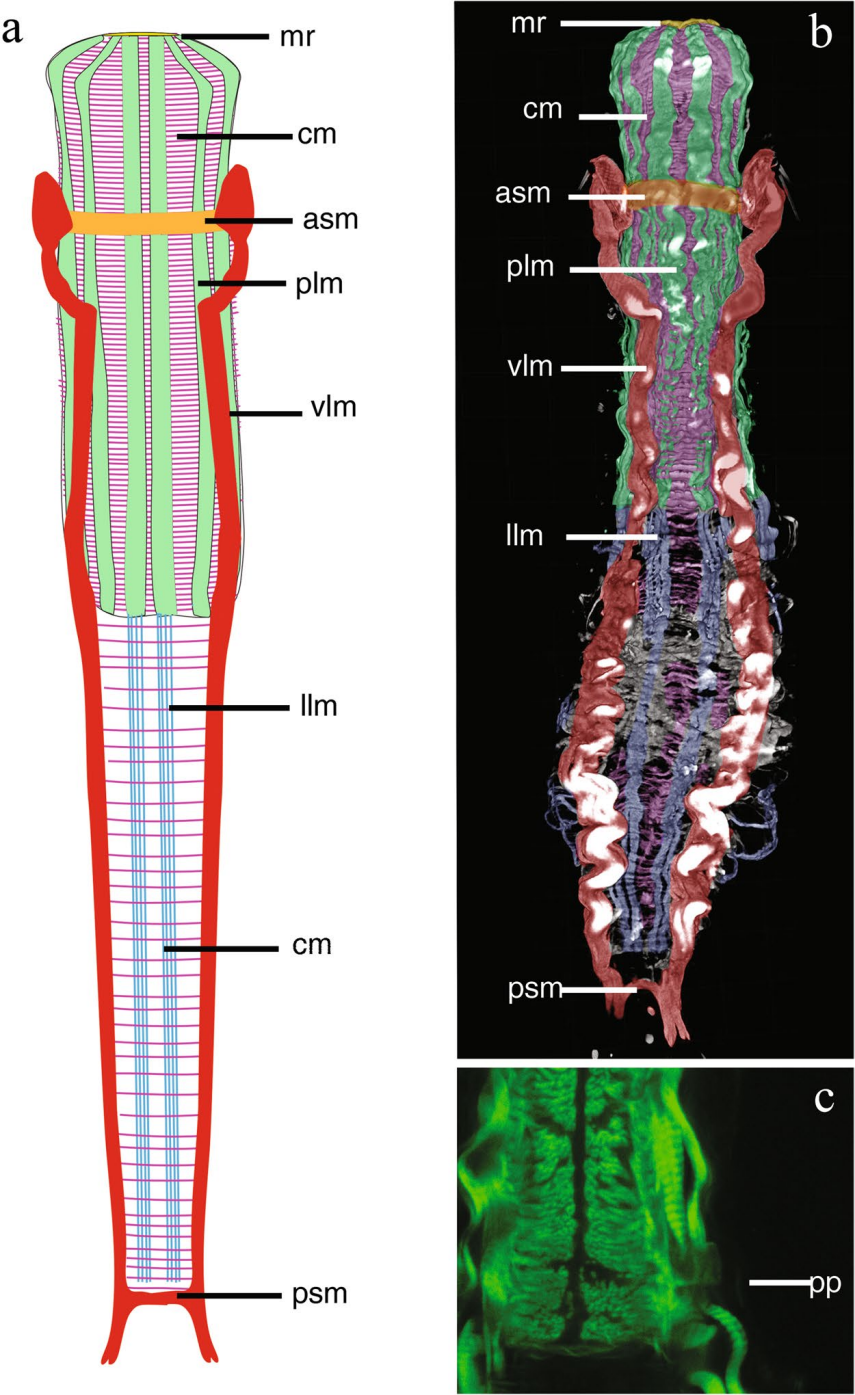
*Redudasys fornerise*. Reconstruction of the musculature from confocal laser scanning microscopy images. (**a**) Schematic drawing of musculature in ventral view. (**b**) Volocity-rendered 3D view of muscles in ventral view. Confocal micrograph of a phalloidin-stained specimen. (**c**) Detail of the pharynx posterior end with pharyngeal pores. asm = anterior semicircular muscle, cm = circular muscles, llm = lateral longitudinal muscles, mr = mouth ring, plm = pharyngeal longitudinal muscles, pp = pharyngeal pore, psm = posterior semicircular muscle, vlm = ventrolateral muscles.

*Redudasys fornerise* - Todaro *et al*.^8^.

*Redudasys* sp. – Garraffoni *et al*.^11^.

### Material

Three specimens collected in July 07, 2014 in Água Limpa Stream, Jequitinhonha drainage basin, Diamantina, State of Minas Gerais, Brazil, were observed alive and filmed. The specimens are no longer available, but videos were deposited at the Zoological Museum, State University of Campinas, accession numbers ZUEC GCH 51, ZUEC GCH 55 and ZUEC GCH 56. One adult specimen collected in September 15, 2015 in Broa Reservoir, Tietê/Jacarei drainage basin, São Carlos, State of São Paulo, Brazil, prepared for confocal analysis and no longer extant. Two additional adult specimens collected in April 11, 2015 and an another two in September 15, 2015, in Broa Reservoir, São Carlos, State of São Paulo, Brazil, mounted for SEM and kept in the first authors’ collection at State University of Campinas.

### Description

The external and internal morphology are in accordance with the original description^7^ and the subsequent redescription^8^ (Figs 5a–f and 6a–c). It is important to highlight that this species has 2 TbA per side, the inner tube shorter than the outer, with a common base that arises from a ventrolateral furrow (Fig. 5c–e). Here we only focus on the muscular system (U46 to U98) (Fig. 6a–c).

Thin circular muscles, very numerous, surround the pharynx for its whole length (U01 to U45), whereas along the intestinal region (U49 to U93) they are in low number and regularly spaced. Eight large longitudinal pharyngeal muscles are inserted at the mouth ring and extend to U45, externally to circular muscles. A group of 4 thick longitudinal muscles per side, internal to the circular musculature, extend ventrally from the PhJIn (U45) to the body posterior end (U96) (Fig. 6a,b).

Two very large longitudinal muscles arise laterally to the pharynx (U13), at TbA insertion (Fig. 6a,b), turning ventrolaterally at U20 and extending up to the posterior body end. They are connected ventrally by two thick transversal muscles at U13 and at U96 respectively (Fig. 6a,b).

Helicoidal muscles were observed along the pharynx, ending at around the PhIJ (U51), but their insertion point could not be observed.

Ovaries and testes not observed; caudal and frontal organs not observed.

### Phylogeny

**Figure 7:**
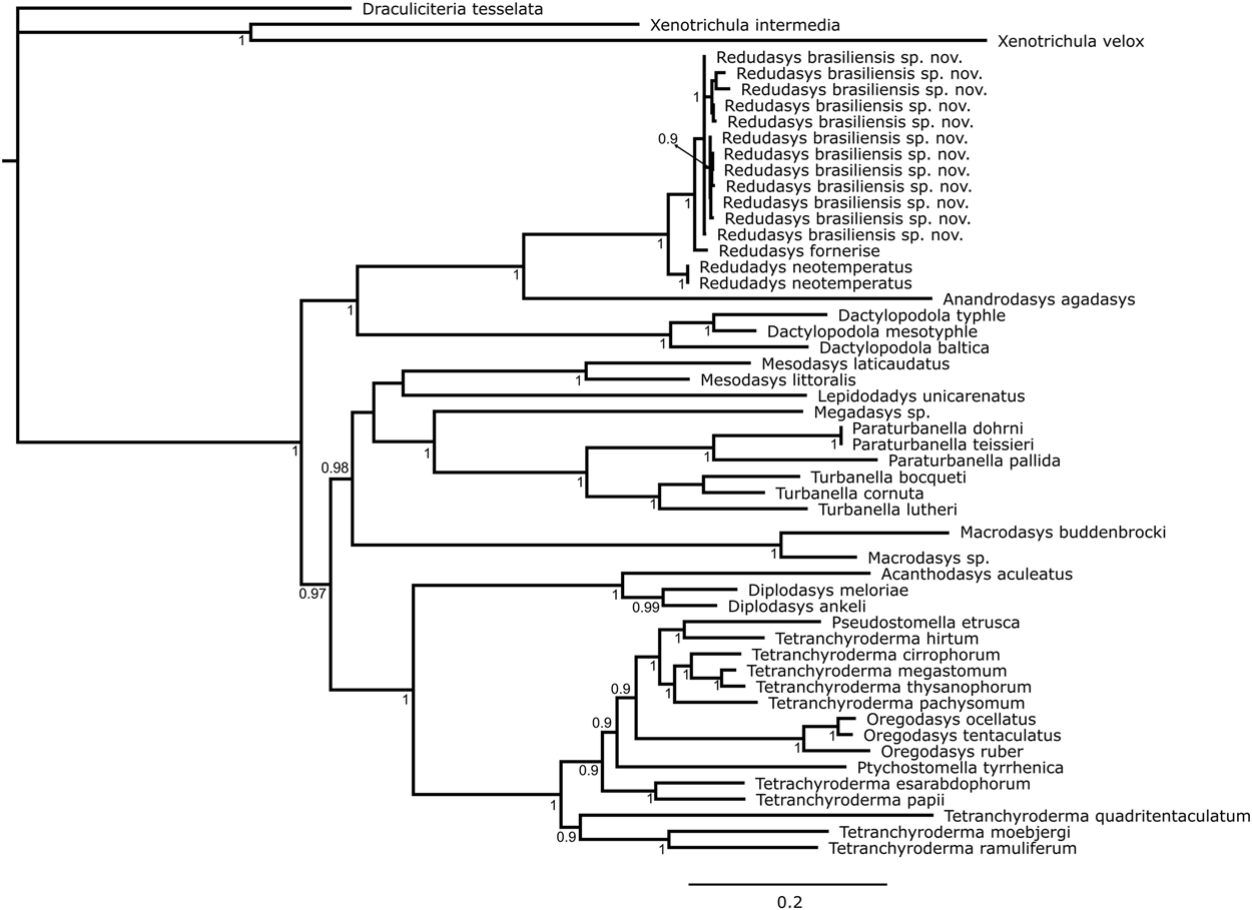
Multigene phylogeny (18S rRNA, 28S rRNA and COI mtDNA) of 37 Gastrotricha species inferred from Bayesian inference analysis. Numbers at nodes represent posterior probabilities.

**Figure 8:**
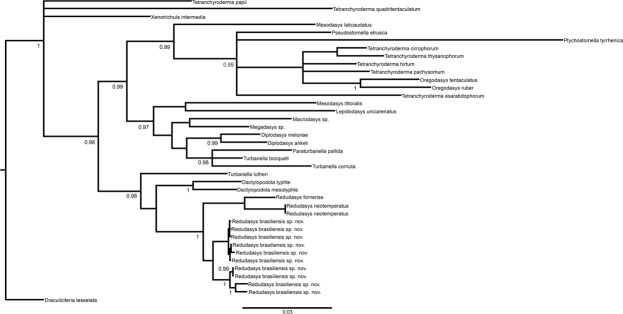
COI mtDNA gene tree of 27 Gastrotricha species inferred from Bayesian inference analysis. Numbers at nodes represent posterior probabilities.

The final alignment of each dataset yielded 2149 positions in 18S rDNA, 4816 positions in 28S rDNA and 730 positions in COI mtDNA). Maximum Likehood (Supplementary Fig. S1) and Bayesian Analysis (Figs 7, 8) analyses yielded congruent topologies. For multigene analysis, the phylogeny showed monophyly of the families Redudasyidae, Thaumastodermatidae, Turbanellidae, Dactylopodolidae, Cephalodasyidae and Macrodasyidae supported in most cases with high bootstrap and Bayesian posterior probability values. Within Redudasyidae, *Redudasys brasiliensis* sp. nov. and *Redudasys fornerise* nested together and this clade formed the sister group of *R. neotemperatus*: all these relations are supported by high bootstrap and Bayesian posterior probability values (respectively, 100 and 1; Supplementary Fig. S1, Fig. 7). The clade with all specimens of *Redudasys brasiliensis* sp. nov. showed high bootstrap (99; Supplementary Fig. S1) but low Bayesian posterior probability (0.6; not shown in Fig. 7) values.

For the analysis of the COI mtDNA, the gene tree only showed the monophyly of the families Dactylopodolidae and Redudasyidae. *Redudasys brasiliensis* sp. nov. resulted as the sister-group of a clade composed by *R. fornerise* and *R*. *neotemperatus*, all supported by high bootstrap and Bayesian posterior probability values. All specimens of *Redudasys brasiliensis* sp. nov. were resolved as a monophyletic group, but with relative low bootstrap (61; not shown in Supplementary Fig. S1) and Bayesian posterior probability values (0.83; not shown in Fig. 8). The specimens of *R. brasiliensis* sp. nov. from São Gonçalo do Rio Preto and from Diamantina formed two distinct monophyletic clades highly supported by bootstrap (respectively, 98 and 97; Supplementary Fig. S1) and Bayesian posterior probability values (respectively, 1 and 1; Fig. 8).

The genetic distance (Table 2) of the haplogroups within each of the two *Redudasys brasiliensis* sp. nov. populations was very low (São Gonçalo do Rio Preto: *p*-distance = 0.3%; Diamantina: *p-*distance = 0.2%) as well as was between the two populations (4.2%). Comparing the values of the two populations with the two other species, the variation was higher: *p*-distances between the Diamantina clade of *R. brasiliensis* and *R. fornerise* ranged from 14.2% to 14.4%; *p*-distances between the São Gonçalo do Rio Preto clade of *R. brasiliensis* and *R. fornerise* ranged from 13.8% to 14.0%; *p*-distances between the Diamantina clade of *R. brasiliensis* and *R. neotemperatus* ranged from 15.6% to 15.8%; *p*-distances between the São Gonçalo do Rio Preto clade of *R. neotemperatus* and *R. fornerise* ranged from 14.6% to 14.8%. Thus, the relation of the maximum within-group genetic distance and the minimum between-group distances supported the identification of four candidate species (*Redudasys brasiliensis* sp. nov. population São Gonçalo do Rio Preto; *R. brasiliensis* sp. nov. population Diamantina; *R. fornerise*; *R. neotemperatus*).

**Table 2:** Average uncorrected *p*-distances among specimens of *Redudasys brasiliensis* sp. nov., *R. fornerise* and *R. neotemperatus* calculated from the COI mtDNA gene alignment.

	1	2	3	4	5	6	7	8	9	10	11
1. *Redudasys brasiliensis* gen. et sp. nov. (São G. do Rio Preto)											
2. *Redudasys brasiliensis* gen. et sp. nov. (São G. do Rio Preto)	0.006										
3. *Redudasys brasiliensis* gen. et sp. nov. (São G. do Rio Preto)	0.002	0.004									
4. *Redudasys brasiliensis* gen. et sp. nov. (São G. do Rio Preto)	0.002	0.004	0.000								
5. *Redudasys brasiliensis* gen. et sp. nov. (Diamantina)	0.077	0.077	0.075	0.075							
6. *Redudasys brasiliensis* gen. et sp. nov. (Diamantina)	0.077	0.077	0.075	0.075	0.002						
7. *Redudasys brasiliensis* gen. et sp. nov. (Diamantina)	0.077	0.077	0.075	0.075	0.002	0.000					
8. *Redudasys brasiliensis* gen. et sp. nov. (Diamantina)	0.079	0.079	0.077	0.077	0.004	0.002	0.002				
9. *Redudasys brasiliensis* gen. et sp. nov. (Diamantina)	0.077	0.077	0.075	0.075	0.002	0.000	0.002	0.002			
10. *Redudasys brasiliensis* gen. et sp. nov. (Diamantina)	0.079	0.079	0.077	0.077	0.004	0.002	0.002	0.004	0.002		
11. *Redudasys fornerise*	0.140	0.140	0.138	0.138	0.144	0.142	0.142	0.144	0.142	0.144	
12. *Redudasys neotemperatus*	0.148	0.148	0.146	0.146	0.158	0.156	0.156	0.158	0.156	0.158	0.180

The parsimony network representing 10 sequences detected a total of seven haplotypes of *Redudasys brasiliensis* sp. nov. (Supplementary Fig. S2). As in the COI mtDNA gene tree, the haplotypes from the two populations were divided into two lineages. The latter species is separated from *R. fornerise* and *R. neotemperatus* by high number of mutations in the network (Supplementary Fig. S2).

## Discussion

In this section, we discuss some morphological and biogeographical patterns that may be associated with the diversification of freshwater macrodasyids.

### Comparison among members of Redudasyidae

Members of family Redudasyidae show few external and internal morphological characters and hence the few characters concerning the adhesive system and locomotory cilia take on systematic significance^12^. Originally^7,8^, the main diagnostic characters of the genus *Redudasys* were the possession of 2 TbA per side and a ventral locomotory ciliation arranged into separate ciliary fields of unequal size (paired in the pharyngeal region and unpaired along the median trunk region). *Anandrodasys*, the only marine genus of the family, shows instead 3 TbA per side and ventral cilia arranged into an unified field posterior to the mouth and splitting into 2 paired longitudinal bands of different length.

Kånneby and Kirk^9^ described the first *Redudasys* species with the presence of a single pair of anterior adhesive tubes, as observed in *R*. *brasiliensis* sp. nov. Garraffoni *et al*.^11^. also reported an unnamed species of *Redudasys* with a single TbA per side from Brazil, but in this case that observation was a misinterpretation. During the present study we had the opportunity to collect fresh material and analyze *Redudasys* specimens from one of the sites previously investigated^11^. This allowed us to verify that these individuals in fact had 2 TbA of unequal size per side arising from a common base just like in *R. fornerise*. Thus, we considered that the *Redudasys* specimens found by Garraffoni *et al*.^11^. can be treated as *R. fornerise*.

The new species can be distinguished from *Redudasys neotemperatus* by: a different pattern of locomotory ciliation (*i*.*e*., two large longitudinal bands close to each other, extending from the mouth ring to the anterior trunk, then merging into a single band up to the caudal body end instead of regularly spaced paired tufts in *R. neotemperatus*), a greater body size (302–376 µm instead of 220–284 µm), a lower number of dorsolateral sensorial cilia (6 pairs instead of 13–14). Furthermore, *Redudasys brasiliensis* sp. nov. is also characterized by pharyngeal pores very poorly developed, unlike the other species of the family.

*Redudasys brasiliensis* sp. nov. and *R. fornerise* possess a quite similar muscular system, despite some specific muscles showing clearly distinct morphological patterns. The circular muscles of *R. brasiliensis* sp. nov. are external to the dorsal longitudinal muscles in the posterior pharyngeal region whereas in *R. fornerise*, they are internal to all longitudinal muscles. Moreover, the main longitudinal body muscles are ventrolateral in *R. brasiliensis* sp. nov. but clearly ventral for almost the whole length in *R*. *fornerise*. Finally, both species show two semicircular transverse muscles connecting the two ventrolateral muscles, but the anterior one is clearly thinner in *R. brasiliensis* sp. nov. than in *R. fornerise*.

The two populations of *Redudasys brasiliensis* sp. nov. were considered two separated species based on “4X rule” approach to delimiting species using DNA sequences^15,17^. However, this result can be biased due to limited number of individuals and candidate species (respectively, 12 and 3–4) and also because two of these 3–4 candidate species (*R. fornerise* and *R. neotemperatus*) had only 1 sequence each available in the GenBank (Supplementary Table S1). Thus, until more specimens of the three species can have their DNA sequenced, we still considered the individuals of both São Gonçalo do Rio Preto and Diamantina populations belonging to the same species, *i.e., R. brasiliensis* sp. nov.

Araújo *et al*.^10^. identified two freshwater macrodasyid specimens found in Brazil as belonging to genus *Marinellina*, basically because they had a single TbA per side. A subsequent study^18^ on the spatial and temporal distribution patterns of freshwater meiofaunal taxa in a lotic system in Brazil found additional macrodasyid specimens with a single TbA per side and reported them as an unidentified species of Redudasyidae. Considering the diagnostic character of the number of TbA, the metric data which fall in the measurement range of *Redudasys brasiliensis* sp. nov., as well as the geographic origin of these specimens, we consider the specimens found in both studies as belonging to the new species. Furthermore, the putative assignment of these specimens to the genus *Marinellina*^10^ cannot be supported with certainty due to several reasons: (a) insufficiency of the original description of the genus *Marinellina*^1,6,7^, (b) uncertainty about the exact position of TbA in *Marinellina* specimens^2^ (c) absence of pharyngeal pores in *Marinellina* specimens, (d) geographic distance between the sampling sites of european individuals and of the specimens found by Araújo *et al*.^10^, respectively.

The new species joins the few other species of macrodasyids that lack a male system including the frontal and caudal accessory organs, useful to store foreign spermatozoa and to act as a copulatory organ – *sensu* Ruppert and Shaw^19^ –, respectively. In these species, that are *Anandrodasys agadasys, Redudasys fornerise*, *R*. *neotemperatus* (Redudasyidae), *Paradasys pacificus* (Cephalodasyidae) and *Urodasys viviparus, Thaidasys tongiorgii* (Macrodasyidae)^20,21^, a probable parthenogenetic condition occurs. Only the latter species has a muscular organ of unknown function, which appears similar in position and structure to the caudal organ of other Macrodasyida^20^.

### Colonization of freshwater habitats by Macrodasyida

Marine Macrodasyida are widely distributed along coastlines of the world and some level of endemism can be found in the North Hemisphere^22^. However, the levels of diversity and endemism of freshwater macrodasyids are considerably lower than in marine ones and, until now, no hypothesis or factor was presented to shed light on this great faunistic heterogeneity.

The Neotropical region seems to be a special area for the speciation of freshwater Macrodasyida. In Brazil, Kisielewski^7^ described the first undoubted macrodasyid from inland waters (*Redudasys fornerise*); subsequently Garraffoni *et al*.^11^ and Araújo *et al*.^10^ reported other freshwater specimens of Macrodasyida and in the present study we describe *Redudasys brasiliensis* sp. nov. These findings highlight the presence and the relatively wide distribution of Macrodasyida in the inland waters of this geographical area.

As pointed out by Ribeiro^23^ the hydrographic basins where populations of *Redudasys fornerise* and *R. brasiliensis* sp. nov. were found have an ancient biogeographic component. The populations of *R. fornerise* from State of São Paulo and the specimens of *R. fornerise* and *R. brasiliensis* sp. nov. from State of Minas Gerais are all very far from the Atlantic shore, respectively around 280 km and 420–450 km. However, these distances were only established during the last sea-level fluctuations in the Holocene transgression, about 5,600 years ago^24,25^. It is important to highlight that the South American continent has not always responded as a single rigid blocks during most of its geological history. The old history of South America shows that this large landmass has an intricate mosaic of amalgamation and break-up of several distinct continental fragments record in different periods^23,26^. Looking into the tectonic-sedimentary evolution of some provinces in the South American Platform, such as areas of São Paulo and Minas Gerais States, it is possible to observe specific orogenic cycles and depositional ages^27–29^. The sampling site in the State of São Paulo is located in the Bauru Basin represented by sandstones and reddish siltstones formed mainly in the Upper Cretaceous (99.6 to 65.5 Mya)^27^. On the other hand, the sampling sites from State of Minas Gerais lie in Lower Espinhaço Basin that is a mountain chain built up mainly of quartzitic rocks formed by a sequence of an intracontinental rift-sag basin system that developed around 1,500 Mya (Mesoproterozoic) to 600 Mya (Neoproterozoic)^28,29^.

These mosaics of smaller microblocks were surrounded by seaway and the current sampling sites could have been much closer to shallow marine environments than today. In this perspective, environment-specific diversification of freshwater macrodasyids within continental waters happened after the incursions of ancestral marine populations. The phylogenetic position of the monophyletic lineages composed by all *Redudasys* species is clearly nested into Macrodasyida clade, suggesting that the freshwater macrodasyids invaded inland environment only once. Furthermore, the phylogenetic hypothesis supports an independent colonization of freshwater habitats by Macrodasyida and Chaetonotida. This scenario is in agreement with the hypothesis advanced by Todaro *et al*.^8^, and in contrast with that proposed by Kieneke *et al*.^4^, implying a single colonization event of freshwater habitats in the stem lineage of Abursata ((*Redudasys*+*Marinellina*) + Paucitubulatina).

Some questions about the biogeographical and evolutionary history of the freshwater Macrodasyida remains unanswered: (a) how did *Redudasy*s lineages achieve their current distributions in independent freshwater systems of Nearctic and Neotropics?; (b) why appear *Redudasys* populations found in freshwater habitats of the Austral hemisphere to be more widespread than those in Boreal hemisphere?; (c) how can the awareness of the existence of *Marinellina*, a taxon *incertae sedis*, affect our current biogeographic and evolutionary understanding of Redudasyidae?

### Faunistic observations

Until now, 76 freshwater species of Gastrotricha from Brazil have been reported^7,8,10,11,30–33^. As highlighted by Garraffoni *et al*.^30^, due to the low number of sampled sites and wide variety of Brazilian freshwater habitats (with distinct geological and abiotic conditions) the number of new species will increase considerably in the next years. As an example for this statement, both the recently described new genus and species *Cephalionotus kisielewskii*^30^ and *Redudasys brasiliensis* sp. nov. are psammic and from the same region, Diamantina Plateau in the Espinhaço Range. The geological structure of this area is composed of high altitude formations (more than 900 m), has a very specific vegetation known as rocky fields (“*campos rupestres*”), and shows special conditions of climate, soil, and water causing high rates of endemisms, especially of plant species^34^.

### Methods

Samples of upper stream sediment were taken from 7 distinct stations (Supplementary Fig. S3): State of Minas Gerais: Jequitinhonha drainage basin - 1. Água Limpa Stream, Biribiri State Park, City of Diamantina (sandy substrate): 18°12′S – 43°37′W; 2. Soberbo Stream, City of Diamantina (sandy/rocky substrate): 18°11′S – 43°33′W; 3. Preto River, Rio Preto State Park, City of São Gonçalo do Rio Preto (sandy/rocky substrate): 18°06′S – 43°20′W; 4. Veado Pool, Rio Preto State Park, City of São Gonçalo do Rio Preto (sandy substrate): 18°06′S – 43°20′W; São Francisco drainage basin - 5. unnamed stream 1 in Sempre-Vivas Federal Park, City of Diamantina (sandy/rocky substrate): 17°50′S – 43°45′W; 6. unnamed stream 2 in Sempre-Vivas Federal Park, city of Diamantina (sandy/rocky substrate): 17°55′S – 43°48′W; State of São Paulo: 7. Broa reservoir, City of São Carlos (sandy substrate): 22°20′S – 47°48′W. The sampling sites 5 and 6 were the same reported by Araújo *et al*.^10^.

Living individuals were located by sorting the sediment poured into Petri dishes under a stereomicroscope Leica EZ4; they were mounted singly on glass slides, observed *in vivo* and anaesthetized with 2% MgCl_2_ under a PrimoStar Zeiss light microscope, and photographed and filmed using a Leica DM2500 microscope equipped with differential interference contrast optics (DIC) and a camera Moticam 2300 of 3.0 megapixel. The videos are available upon request from the first author.

The positions of morphological characters along the body were measured using the percentage units (U)^35^.

### Scanning Electron Microscopy

For Scanning Electronic Microscopy (SEM) analysis, the specimens were fixed in 4% formalin, dehydrated through a graded series of ethanol, treated with HMDS (hexamethyldisilazane)^36^, mounted onto aluminum stub and coated with gold-palladium using sputter coating. Observations were carried out under a SEM JSM 5800LV, at the State University of Campinas (Brazil), and a SEM Philips 515, at the University of Urbino (Italy).

### Transmission Electron Microscopy

For Transmission Electronic Microscopy (TEM) analysis, the specimens were fixed in 2% glutaraldehyde in a 0.1 M sodium cacodylate buffer (pH 7.4), post-fixed in 1% osmium tetroxide buffered with 0.1 M sodium cacodylate, washed in a clean 0.1 M sodium cacodylate buffer, dehydrated in a graded ethanol series and embedded in Araldite. Semi-thick and ultra-thin sections were cut with a LKB Ultrotome 2088 V microtome and contrasted with toluidine blue and lead citrate, respectively. The semi-thin sections were observed in transmission light under a VANOX AHBT3 Olympus optical microscope, whereas the ultra-thin sections (70–80 nm) were observed under a TEM Philips CM10 at the University of Urbino (Italy).

### Confocal Laser Scanning Microscopy

For Confocal Laser Scanning Microscopy (CLSM) the specimens were fixed in 4% paraformaldehyde in 0.1 M phosphate buffer saline (pH 7.2) for at least one week. Specimens were then rinsed in PBS and stained with Alexa Fluor 488 phalloidin (Life Technologies) to document the musculature. Stained specimens were briefly rinsed in PBS before mounting in Fluoromount G (Southern Biotechnology Associates, Birmingham, AL) on glass slides. An Olympus FV 300 confocal laser-scanning microscope was used to observe the specimens at the State University of Campinas (Brazil). An Argon laser (488 nm) was used to excite the samples, and Olympus software was used to capture the images. Confocal z-stacks were collected and processed as TIF files and MOV video files. Files were further processed with the software Volocity (Perkin Elmer) to generate z-projections.

### DNA extraction and PCR amplification

Genomic and mitochondrial DNA was extracted from entire individuals of *Redudasys brasiliensis* sp. nov. using a column-based method QIAamp DNA Micro Kit (Qiagen), following the manufacturer’s instructions. DNA samples were subjected to PCR amplifications in a reaction volume of 25 µL containing 12.5 µL of 2x Taq PCR Master Mix (Qiagen), 3 µL of DNA, 8.7 µL of water, and 0.4 µL (4 pmol) of specific primers. The primer sequences and PCR conditions are indicated in Supplementary Table S2. The amplification products were electrophoresed in 1% agarose gels containing SYBR Green (Life Technologies). The bands with expected sizes were excised and then purified using a QIAquick Gel Extraction Kit (Qiagen) or the PCR product was directly purified using Illustra ExoProStar 1-Step (GE Healthcare). The DNA fragments were sequenced using BigDye Terminator reactions in a 3730XL DNA Analyzer (Applied Biosystems) at the facility of the LaCTAD laboratory (Campinas, Brazil). The 18S rDNA, 28S rDNA and COI mtDNA partial DNA sequences of *Redudasys brasiliensis* sp. nov. were deposited in GenBank (Supplementary Table S2).

### Sequences, alignments and data analyses

18S rDNA, 28S rDNA and COI mtDNA sequences were aligned separately with Mafft v.7.215 using the L-INS-I approach^37^. The best-fit substitution model was determined with jModelTest 2.1.4^38^. As the number of species analyzed with three genes and deposited in the GenBank is low, we run two distinct analyses (combined dataset and only COI) under maximum likelihood (ML) and Bayesian inference methods (BA) frameworks. ML analysis using RAxML^39^ was run with a GTRCAT model with 1000 bootstrap replicates. BA analysis was done using MrBAYES v.3.2.3^40^ using two different runs with four chains each for a maximum of 20 million generations (sampled every 500 generations). Best-fit evolutionary model was selected using Akaike Information Criterion. The analysis was stopped when the two runs reached convergence (average standard deviation of split frequencies lower than 0.01). Convergence and estimated sample size (ESS) were verified using TRACER v.1.5, and 10% of each run was discarded as burn-in. Both ML and BA analyses were performed using the CIPRES Science Gateway, San Diego Supercomputer Center^41^.

For combined dataset, sequences of 37 species were retrieved from GenBank (2 Cephalodasyidae; 3 Dactylopodolidae; 2 Macrodasyidae; 1 Planodasyidae; 1 Lepidodasyidae; 18 Thaumastodermatidae; 6 Turbanellidae; 4 Redudasyidae) and for COI mtDNA, sequences of 27 species deposited in GenBank were included in the analysis (2 Cephalodasyidae; 3 Dactylopodolidae; 1 Macrodasyidae; 1 Lepidodasyidae; 13 Thaumastodermatidae; 4 Turbanellidae; 3 Redudasyidae) (Supplementary Table S2). Three species belonging to the order Chaetonotida, family Xenotrichulidae: *Draculiciteria tesselata* (Renaud Mornant, 1968), *Xenotrichula intermedia* Remane, 1934 and *X*. *velox* Remane, 1927 were used as outgroups (Supplementary Table S2). These species were selected as outgroups because members of Xenotrichulidae appeared as the sister group of all other Chaetonotida (e.g. Kånneby *et al*.^42^).

### Genetic distances

MEGA7 software^43^ was used to calculate average uncorrected pairwise distances (*p*-distances) within and between species and haplogroups; alignment gaps were not considered. Moreover, the reliability of the new species diversification was tested applying the K/ϴ model (formerly known as 4X rule, Birky^17^, Fontaneto *et al*.^15^).

### Statistical parsimony networks

To visualize possible intraspecific relationships between the COI haplotypes for *Redudasys fornerise*, *R*. *neotemperatus* and *Redudasys brasilensis* sp. nov., statistical parsimony networks were calculated using the program PopART^44^. The raw networks produced by PopART were redrawn using software Adobe Photoshop CS6 (Supplementary Fig. S2).

## Supplementary information

Supplementary information
