# Supplementary material for: Integrative taxonomy of a new Redudasys species (Gastrotricha: Macrodasyida) sheds light on the invasion of fresh water habitats by macrodasyids
Source: Sci Rep. 2019 Feb 14;9:2067. doi: 10.1038/s41598-018-38033-0 (PMC6376054; doi:10.1038/s41598-018-38033-0)
Supplement: Supplementary file 1 — Supplementary information [file 41598_2018_38033_MOESM1_ESM.docx]

Integrative taxonomy of a new *Redudasys* species (Gastrotricha: Macrodasyida) sheds light on the invasion of fresh water habitats by macrodasyids

André R.S. Garraffoni^1^, Thiago. Q. Araújo^2^, Anete P. Lourenço^3^, Loretta Guidi^4^ and Maria Balsamo^4^

^1^Departament of Animal Biology, Institute of Biology, State University of Campinas, R. Monteiro Lobato, 255, 13083-970, Campinas, SP, Brazil. ^2^Departament of Zoology, Institute of Biological Science, Federal University of Minas Gerais, Av. Antonio Carlos, 6627, 31270-901, Belo Horizonte, MG, Brazil. ^3^Departament of Biological Science, Federal University of Jequitinhonha and Mucuri Valleys, Rodovia BR-367, 39100-000, Diamantina, MG, Brazil. ^4^Department of Biomolecular Sciences, University of Urbino, Campus Scientifico, Via Ca’ le Suore, 2, 61049, Urbino, PU, Italy. Correspondence and requests for materials should be addressed to A.R.S.G. (email: arsg@unicamp.br)

Table 2S. Taxa included in this study, with GenBank accession numbers of 18S rDNA, 28S rDNA and COI mtDNA sequences.

| **Taxon** | **18S** | **28S** | **COI** | **References** |
| --- | --- | --- | --- | --- |
| **Order Macrodasyida** |  |  |  |  |
| Family Cephalodasyidae |  |  |  |  |
| *Mesodasys laticaudatus* | JF357657 | JF357705 | JF432050 | Todaro et al.^1^ |
| *Mesodasys littoralis* | JF357658 | JF357706 | JF432044 | Todaro et al.^1^ |
| Family Dactylopodolidae |  |  |  |  |
| *Dactylopodola baltica* | KC193098 | KC193099 | JN003637 | Golombek et al.^2^; Kieneke et al^3^ |
| *Dactylopodola mesotyphle* | JF357651 | JF357699 | JF432036 | Todaro et al.^1^ |
| *Dactylopodola typhle* | JF357653 | JF357701 | JF432038 | Todaro et al.^1^ |
| Family Macrodasyidae |  |  |  |  |
| *Macrodasys buddenbrocki* | AY963692 | KF92101 | - | Petrov et al.^4^ |
| *Macrodasys* sp. | JF357654 | JF357702 | JF432040 | Todaro et al.^1^ |
|  |  |  |  |  |
| Family Planodasyidae |  |  |  |  |
| *Megadasys* sp. | JF357655 | JF357703 | JF432042 | Todaro et al.^1^ |
| Family Lepidodasyidae |  |  |  |  |
| *Lepidodasys unicarenatus* | JF357665 | - | JF432039 | Todaro et al.^1^ |
| Family Thaumastodermatidae |  |  |  |  |
| *Acanthodasys aculeatus* | JF357639 | JF357687 | - | Todaro et al.^1^ |
| *Diplodasys meloriae* | JF357632 | JF357688 | JF432031 | Todaro et al.^1^ |
| *Diplodasys ankeli* | JF357624 | - | JF432049 | Todaro et al.^1^ |
| *Pseudostomella etrusca* | JF357633 | JF357681 | JF432026 | Todaro et al.^1^ |
| *Ptychostomella tyrrhenica* | JF357634 | JF357682 | JF432027 | Todaro et al.^1^ |
| *Tetranchyroderma cirrophorum* | JF357635 | JF357683 | JF432028 | Todaro et al.^1^ |
| *Tetranchyroderma esarabdophorum* | JF357627 | - | JF432022 | Todaro et al.^1^ |
| *Tetranchyroderma hirtum* | JF357628 | JF357676 | JF432023 | Todaro et al.^1^ |
| *Tetranchyroderma megastomum* | KC193100 | KC193101 | - | Golombek et al.^2^ |
| *Tetranchyroderma pachysomum* | JF357636 | JF357684 | JF432029 | Todaro et al.^1^ |
| *Tetranchyroderma papii* | JF357637 | JF357685 | JF432030 | Todaro et al.^1^ |
| *Tetranchyroderma quadritentaculatum* | JF357647 | JF357695 | JF432024 | Todaro et al.^1^ |
| *Tetranchyroderma thysanophorum* | JF357646 | JF357694 | JF432025 | Todaro et al.^1^ |
| *Thaumastoderma moebjergi* | JF357671 | JF357713 | - | Todaro et al.^1^ |
| *Thaumastoderma ramuliferum* | JF357649 | JF357679 | - | Todaro et al.^1^ |
| *Oregodasys ocellatus* | JF357642 | JF357690 | - | Todaro et al.^1^ |
| *Oregodasys ruber* | JF357625 | JF357673 | JF432020 | Todaro et al.^1^ |
| *Oregodasys tentaculatus* | JF357626 | JF357674 | JF432021 | Todaro et al.^1^ |
| Family Turbanellidae |  |  |  |  |
| *Paraturbanella dohrni* | JF357659 | JF357707 | - | Todaro et al.^1^ |
| *Paraturbanella pallida* | JF357660 | JF357708 | JF432045 | Todaro et al.^1^ |
| *Paraturbanella teissieri* | JF357661 | JF357709 | - | Todaro et al.^1^ |
| *Turbanella bocqueti* | JF357662 | JF357710 | JF432046 | Todaro et al.^1^ |
| *Turbanella cornuta* | JF357666 | JF357711 | JF432047 | Todaro et al.^1^ |
| *Turbanella lutheri* | JF357669 | - | JF432051 | Todaro et al.^1^ |
| Family Redudasyidae |  |  |  |  |
| *Anandrodasys agadasys* | JN203487 | - | - | Todaro et al.^5^ |
| *Redudasys fornerise* | JN203489 | MF577024 | KJ950122 | Todaro et al.^5^; Kånneby & Kirk^6^ |
| *Redudasys* *neotemperatus* | KJ950121 | MF577025 | KJ950123 | Kånneby & Kirk^6^;  Kånneby & Wicksten^7^ |
| *Redudasys* *neotemperatus* | MF577023 | MF577026 | - | Kånneby & Kirk^6^ |
| *Redudasys brasiliensis* sp. nov. | MH361306 | - | - | this study |
| *Redudasys brasiliensis* sp. nov. | MH361307 | - | MH370128 | this study |
| *Redudasys brasiliensis* sp. nov. | MH361308 | - | MH370129 | this study |
| *Redudasys brasiliensis* sp. nov. | MH361309 | - | - | this study |
| *Redudasys brasiliensis* sp. nov. | - | - | MH370130 | this study |
| *Redudasys brasiliensis* sp. nov. | - | MH361314 | MH370131 | this study |
| *Redudasys brasiliensis* sp. nov. | - | MH361315 | MH370132 | this study |
| *Redudasys brasiliensis* sp. nov. | - | - | MH370133 | this study |
| *Redudasys brasiliensis* sp. nov. | MH361310 | MH361316 | MH370134 | this study |
| *Redudasys brasiliensis* sp. nov. | MH361311 | MH361317 | MH370135 | this study |
| *Redudasys brasiliensis* sp. nov. | MH361312 | MH361318 | MH370136 | this study |
| *Redudasys brasiliensis* sp. nov. | MH361313 | MH361319 | MH370137 | this study |
| **Order Chaetonotida** |  |  |  |  |
| Family Xenotrichulidae |  |  |  |  |
| *Draculiciteria tesselata* | JN185457 | JN185506 | JN185541 | Kånneby et al.^8^ |
| *Xenotrichula intermedia* | JF357664 | - | JF432048 | Kånneby et al.^8^ |
| *Xenotricula velox* | JN185499 | JQ798652 | - | Kånneby et al.^8^ |

Table 2S. Primer sequences and PCR regimes used for 18S rDNA, 28S rDNA and COI mtDNA amplification and sequencing.

| **Primers & PCR regimes** | **Primer sequence (5’→3’)** | **Reference** |
| --- | --- | --- |
| **18S primers** |  |  |
| S30 | GCTTGTCTCAAAGATTAAGCC | Norén & Jondelius^9^ |
| S30R | CTTCGGACCTCTGACTTTCG | Garraffoni et al.^10^ |
| PCR S30/S30R | 94^o^C for 5 min, 40x (94^o^C for 30 s, 52.5^o^C for 30 s, 72^o^C for 60 s), 72^o^C for 7 min |  |
| 1801 | GATCTATTTTGTTGGTTTTCGG | Garraffoni et al.^10^ |
| 1806 | CCTTGTTACGACTTTTACTTCCTC | Norén & Jondelius^9^ |
| PCR 1801/1806 | 94^o^C for 5 min, 40x (94^o^C for 30 s, 52.5^o^C for 30 s, 72^o^C for 60 s), 72^o^C for 7 min |  |
| **28S primers** |  |  |
| 28S.1F | CCTAAAGTAACGGCGAGTGA | This study |
| 28S.1R | CGATTAGTCTTTCGCCCCTA | This study |
| PCR 28S.1F/28S.1F | 94^o^C for 5 min, 40x (94^o^C for 30 s, 55^o^C for 30 s, 72^o^C for 60 s), 72^o^C for 7 min |  |
| 28S.2F | GGACCCGAAAGATGGTGAAC | This study |
| 28S.2R | CAATTTGCCGACTTCCCTTG | This study |
| PCR 28S.2F/28S.2F | 94^o^C for 5 min, 40x (94^o^C for 30 s, 60^o^C for 30 s, 72^o^C for 60 s), 72^o^C for 7 min |  |
| **COI primers** |  |  |
| LCO1490 | GGTCAACAAATCATAAAGATATTGG | Folmer et al.^11^ |
| HCO2198 | TAAACTTCAGGGTGACCAAAAAATCA | Folmer et al.^11^ |
| PCR LCO1490/ HCO2198 | 94^o^C for 5 min, 40x (94^o^C for 30 s, 46^o^C for 30 s, 72^o^C for 40 s), 72^o^C for 7 min |  |

1. Todaro, M. A., Kånneby, T., Dal Zotto, M. & Jondelius, U. Phylogeny of Thaumastodermatidae (Gastrotricha: Macrodasyida) inferred from nuclear and mitochondrial sequence data. *PLoS One* **6:** E17892 (2011).

2. Golombek, A., Tobergte, S., Nesnidal, M. P., Purschke, G., & Struck, T. H. Mitochondrial genomes to the rescue–Diurodrilidae in the myzostomid trap. *Mol. Phylogenet. Evol.* **68**: 312-326 (2013).

3. Kieneke, A., P. M. Martinez-Arbizu & D. Fontaneto, Spatially structured populations with a low level of cryptic diversity in European marine Gastrotricha. *Mol. Ecol.* **21**: 1239–1254 (2012).

4. Petrov, N. B., Pegova, A. N., Manylov, O. G., Vladychenskaya, N. S., Mugue, N. S., & Aleshin, V. V. Molecular phylogeny of Gastrotricha on the basis of a comparison of the 18S rRNA genes: Rejection of the hypothesis of a relationship between Gastrotricha and Nematoda. *Mol. Biol.* **41**: 445-452 (2007).

5. Todaro, M. A., Dal Zotto, M., Jondelius, U., Hochberg, R., Hummon, W. D., Kånneby, T. & Rocha C. E. F. Gastrotricha: A Marine Sister for a Freshwater Puzzle. *PLoS One* 7: e31740 (2012).

6. Kånneby, T., & Kirk, J. J. A new species of *Redudasys* (Gastrotricha: Macrodasyida: Redudasyidae) from the United States. *P. Biol. Soc. Wash.*, **130**: 128-139. (2017)

7. Kånneby, T. & Wicksten, M. K. First record of the enigmatic genus *Redudasys* Kisielewski, 1987 (Gastrotricha: Macrodasyida) from the Northern hemisphere. *Zoosystema* **36**: 723-733 (2014).

8. Kånneby, T., Todaro, M. A., & Jondelius, U. A phylogenetic approach to species delimitation in freshwater Gastrotricha from Sweden. *Hydrobiologia* **683**: 185-202 (2012)

9. Norén, M. & Jondelius U. Phylogeny of the Prolecithophora (Platyhelminthes) inferred from 18S rDNA sequences. *Cladistics* **15**: 103–112 (1999).

10. Garraffoni, A. R., Araújo, T. Q., Lourenço, A. P., Guidi, L., & Balsamo, M. A new genus and new species of freshwater Chaetonotidae (Gastrotricha: Chaetonotida) from Brazil with phylogenetic position inferred from nuclear and mitochondrial DNA sequences. *Syst. Biodivers.* **15**: 49-62 (2017).

11. Folmer, O., Black. M., Hoeh, W., Lutz, R. & Vrijenhoek R. DNA primers for amplification of mitochondrial cytochrome c oxidase subunit I from diverse metazoan invertebrates. *Mol. Mar. Biol. Biotech.* **3**: 294–299 (1994).

a


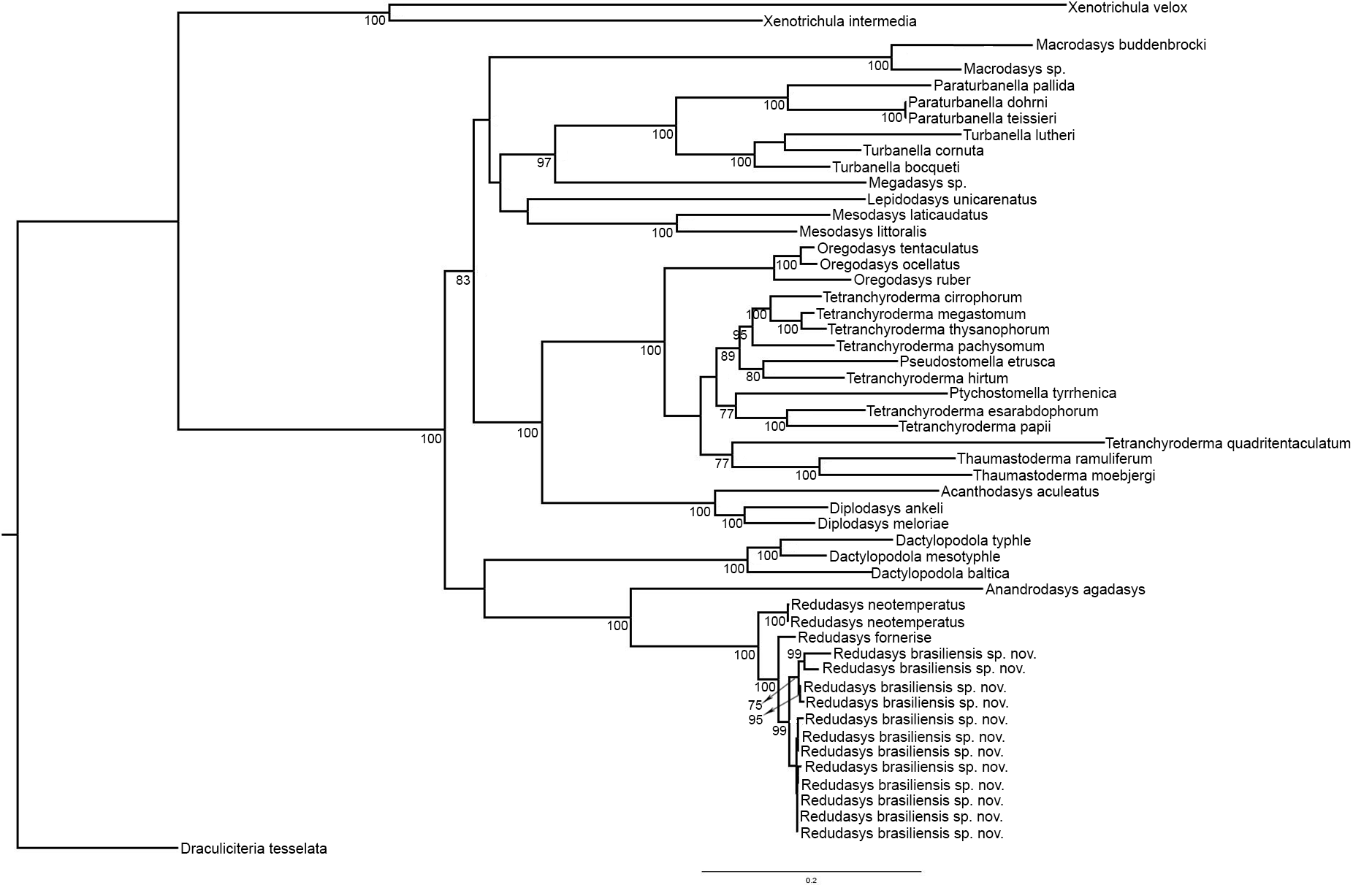


b


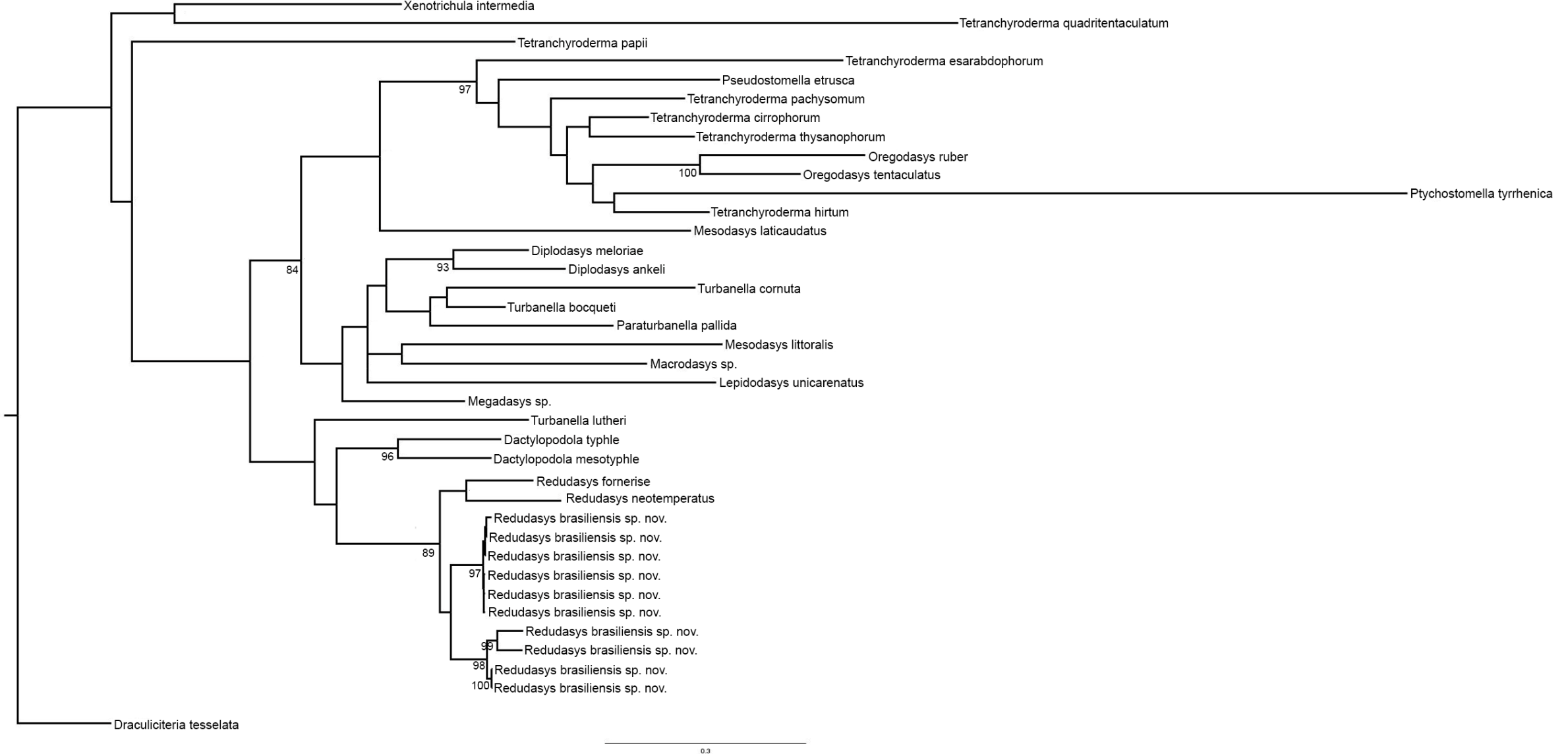


**Figure S1.** Multigene phylogeny (18S rRNA, 28S rRNA and COI mtDNA) (A) and COI gene tree (B) of 40 and 29 Gastrotricha species, respectively, inferred from Maximum Likelihood analysis. Numbers at nodes represent bootstrap support (1000 bootstrap replicates).

**
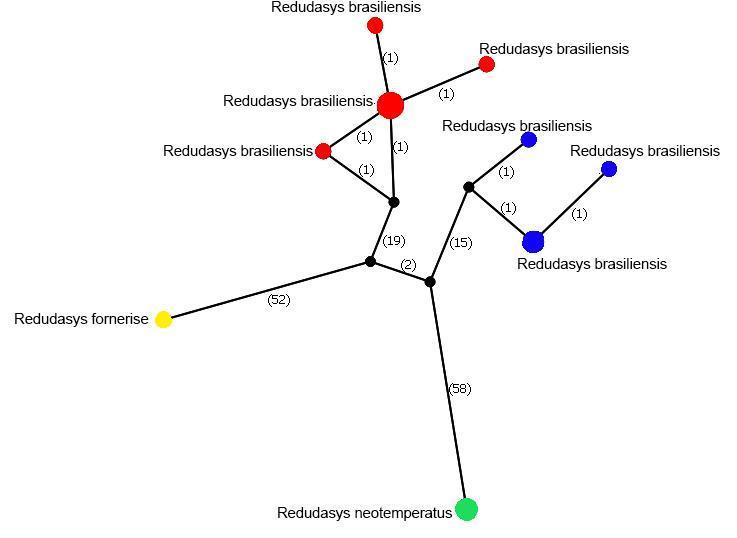
**

**Figure S2.** Statistical parsimony network of *Redudasys brasiliensis* sp. nov., *R*. *fornerise* and *R*. *neotemperatus* haplotypes. Blue dots indicate Rio Preto populations, Red dot indicate Diamantina populations, yellow dot indicates *R*. *fornerise* population, green dot indicates *R*. *neotempertaus* population and black dot indicates missing connecting haplotypes, either not sampled or extinct. Black bars with numbers denote multiple missing haplotypes.


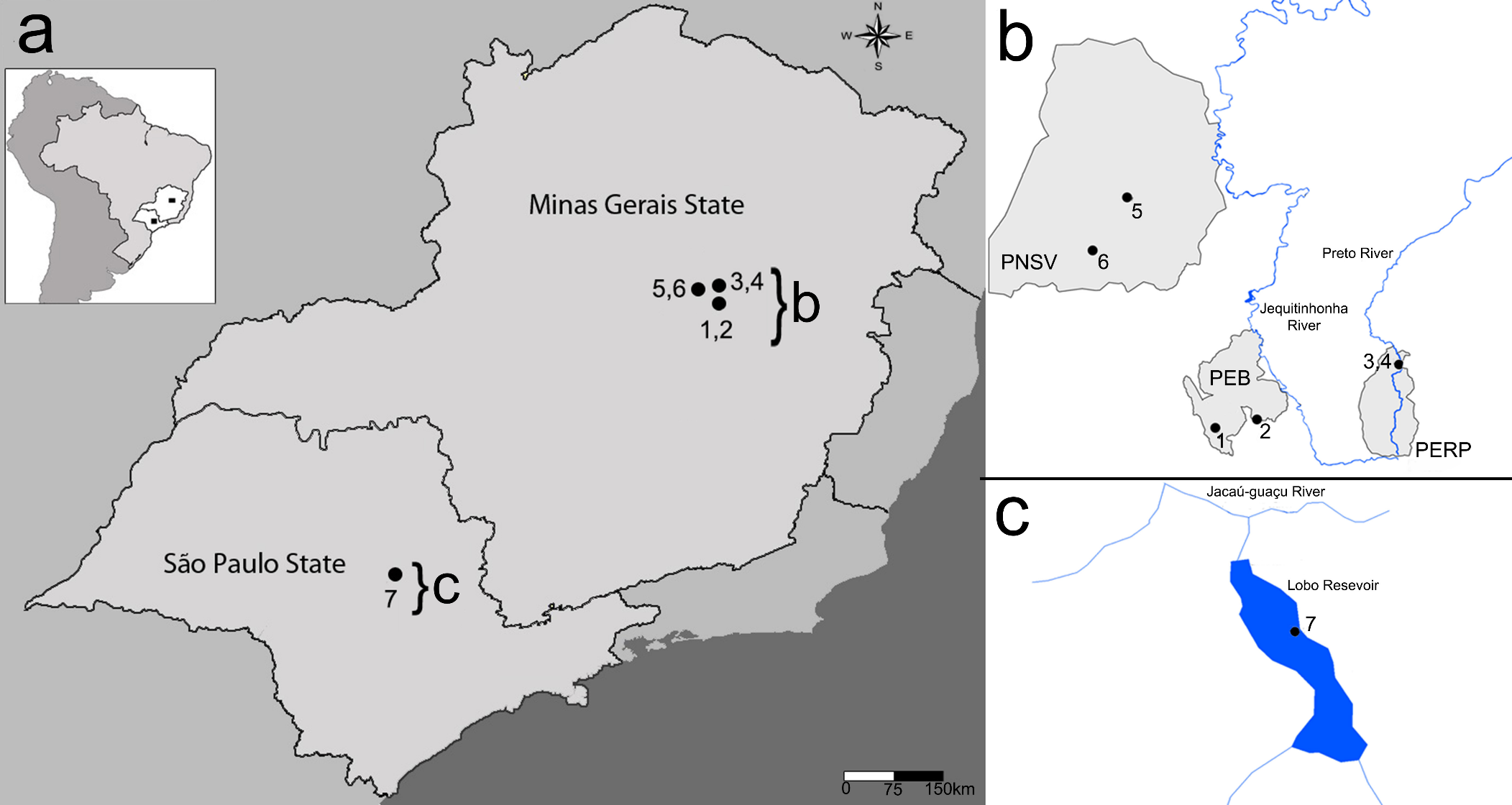


**Figure S3.** A. Localities in the States of Minas Gerais and São Paulo (Brazil) where the specimens were found. B. *Redudasys brasiliensis* sp. nov., PNSV: Parque Nacional da Sempre Viva (Sempre Viva National Park), PEB: Parque Estadual do Biribiri (Biribiri State Park), PERP: Parque Estadual do Rio Preto (Rio Preto State Park); C. *Redudasys fornerise*, Broa Reservoir. Numbers represent the sampling sites in this study, listed in the Materials and Methods. Map data from GPS TrackMaker.
